# Alpha-fetoprotein inhibits autophagy to promote malignant behaviour in hepatocellular carcinoma cells by activating PI3K/AKT/mTOR signalling

**DOI:** 10.1038/s41419-018-1036-5

**Published:** 2018-10-09

**Authors:** Shanshan Wang, Mingyue Zhu, Qiaoyun Wang, Yuli Hou, Lei Li, Honglei Weng, Yan Zhao, Dexi Chen, Huiguo Ding, Junli Guo, Mengsen Li

**Affiliations:** 1 Beijing Institute of Hepatology, Beijing Youan Hospital, Capital Medical University, Beijing, China; 2 Hainan Provincial Key Laboratory of Carcinogenesis and Intervention, Hainan Medical College, Haikou, China; 3 Beijing Precision Medicine and Transformation Engineering Technology Research Center of Hepatitis and Liver Cancer, Beijing, China; 4 Clinical Laboratory Center, Beijing Youan Hospital, Capital Medical University, Beijing, China; 5 Department of Gastrointestinal and Hepatology, Beijing Youan Hospital, Capital Medical University, Beijing, China; 6 Molecular Hepatology, University of Heidelberg, University Medical Center Mannheim, 68167 Mannheim, Germany

## Abstract

Alpha-fetoprotein (AFP) has been recognized as a key regulator of cell proliferation in hepatocellular carcinoma (HCC). However, whether AFP functions in cancer cell autophagy remains unknown. This study investigated the effects of AFP on autophagy in HCC cells. The role of AFP was studied in two HCC cell lines, PLC/PRF/5 and HLE. Cell autophagy, apoptosis, proliferation, migration and invasion were analysed with Western blotting, co-immunoprecipitation (CoIP), immunofluorescence, animal models, MTT assays, flow cytometry (FCM), Cell Counting Kit (CCK)-8, and scratch and transwell assays. In PLC/PRF/5 cells, AFP interacted with PTEN and activated PI3K/Akt/mTOR signalling. In HLE cells, overexpressed AFP similarly interacted with PTEN, leading to PI3K/Akt/mTOR activation and reduced cell autophagy. When AFP was silenced in PLC/PRF/5 cells, cell proliferation, tumour growth, migration and invasion were inhibited, and the numbers of S-phase and apoptotic cells were increased. In contrast, AFP overexpression in HLE cells enhanced cell proliferation, migration and invasion and reduced apoptosis. AFP-dependent autophagy, proliferation, migration and apoptosis were inhibited by rapamycin. In summary, AFP plays critical roles in the inhibition of autophagy and apoptosis in HCC cells and promotes proliferation, migration and invasion. The role of AFP in autophagy inhibition in HCC cells may involve the activation of PI3K/Akt/mTOR signalling.

## Introduction

Autophagy is an important lysosomal process, in which the degradation of cellular components serves to maintain cellular function and survival^1^. Autophagy may determine cell fate through complex signalling pathways and plays an important role in the pathophysiology of the liver. Thus, liver function is highly dependent on autophagy^2^. Such dependence on autophagy is especially notable in several pathological liver diseases, such as hepatitis, alcohol/non-alcoholic fatty liver disease, drug-induced liver injury, and ischaemic injury^3,4^. Autophagy can regulate the proliferation and apoptosis of liver cells in different contexts, but its role in hepatocellular carcinoma cancer (HCC) is controversial^5^. Autophagy is a complex process that involves many signalling pathways. As is currently well known, the PI3K/Akt/mTOR pathway plays an important role in promoting cell autophagy^6^.

Due to its highly aggressive behaviour and high fatality rate, HCC is currently the fifth most common malignancy in the world and is particularly prevalent in China^7^. Alpha-fetoprotein (AFP) is well known for its wide clinical use in the diagnosis and treatment of liver cancer^8^. Over the last ten years, we performed a series of studies to explore other functions of AFP. Based on clinical data, higher AFP levels correlate with higher mortality rates in HCC patients^9^. Multiple lines of evidence show that AFP can function as a growth regulator by binding to key proteins involved in signalling pathways. Subsequent studies have shown that AFP can block RA-RAR signalling to disrupt the forward transmission of apoptotic signalling^10,11^. Furthermore, cytoplasmic AFP interacts with PTEN to activate the PI3K/AKT pathway, leading to aberrant growth and migration of HCC cells^12–15^.

As mentioned above, the evidence that intracellular AFP acts as a signalling regulator and affects HCC growth, apoptosis, cell cycle, and migration is convincing. Therefore, understanding whether intracellular AFP influences autophagy in HCC cells is of particular interest. Our recent experimental results indicated that changes in AFP expression can affect the expression of the cellular autophagy-related protein mTOR, which is involved in the PI3K/AKT pathway^16,17^. Although the underlying mechanisms through which AFP affects cell autophagy remain unclear, the available evidence suggests that AFP plays a major role in autophagy.

The present study aimed to assess the involvement of intracellular AFP in PI3K/Akt/mTOR pathway activation and to provide experimental support for its regulatory properties in autophagy, which have been ascribed to cytoplasmic AFP with respect to the malignant behaviour of HCC cells.

## Results

### Interaction between AFP and PTEN in HCC cells

**Fig. 1:**
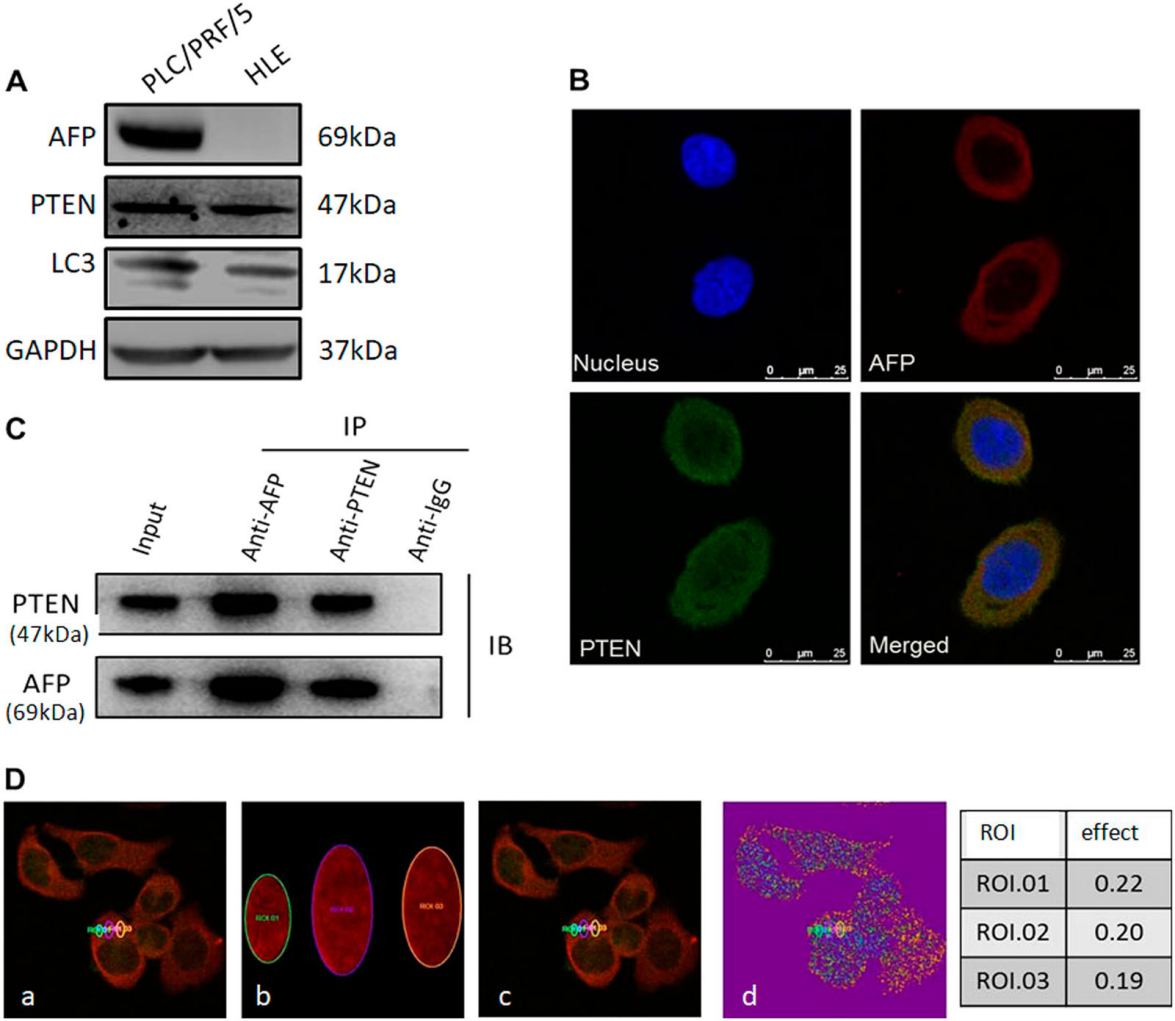
Expression of AFP and its interaction with PTEN in PLC/PRF/5 and HLE cells. **a** Western blotting of AFP, PTEN and LC3 expression in PLC/PRF/5 and HLE cells. **b** Co-localization of AFP and PTEN in PLC/PRF/5 cells. Localization of AFP and PTEN was observed on a laser scanning confocal microscope. Nuclei were stained with DAPI (blue). AFP and PTEN were labelled with TRITC (red) and FITC (green), respectively. **c** The interaction of AFP and PTEN was analysed using co-immunoprecipitation. Lysates from PLC/PRF/5 cells were immunoprecipitated (IP) with antibodies against AFP or PTEN and separated by SDS/PAGE. **d** FRET measurement for Alexa 568-AFP (Donor) and Alexa 488-PTEN (Acceptor) in PLC/PRF/5 cells. (a) Pre-bleaching; (b) Bleaching; (c) Post-bleaching; (d) Bleaching results; Right panel: Bleaching efficiency. All images are representative of at least three independent experiments

Western blotting analysis showed that AFP protein was undetectable in HLE cells but was robustly expressed in PLC/PRF/5 cells (Fig. 1a). Laser scanning confocal microscopy demonstrated that AFP and PTEN colocalized in the cytoplasm of PLC/PRF/5 cells (Fig. 1b). This finding was further confirmed by CoIP (Fig. 1c), as well as by Fluorescence resonance energy transfer(FRET) (Fig. 1d).

### Effects of AFP on PI3K/Akt/mTOR signalling

**Fig. 2:**
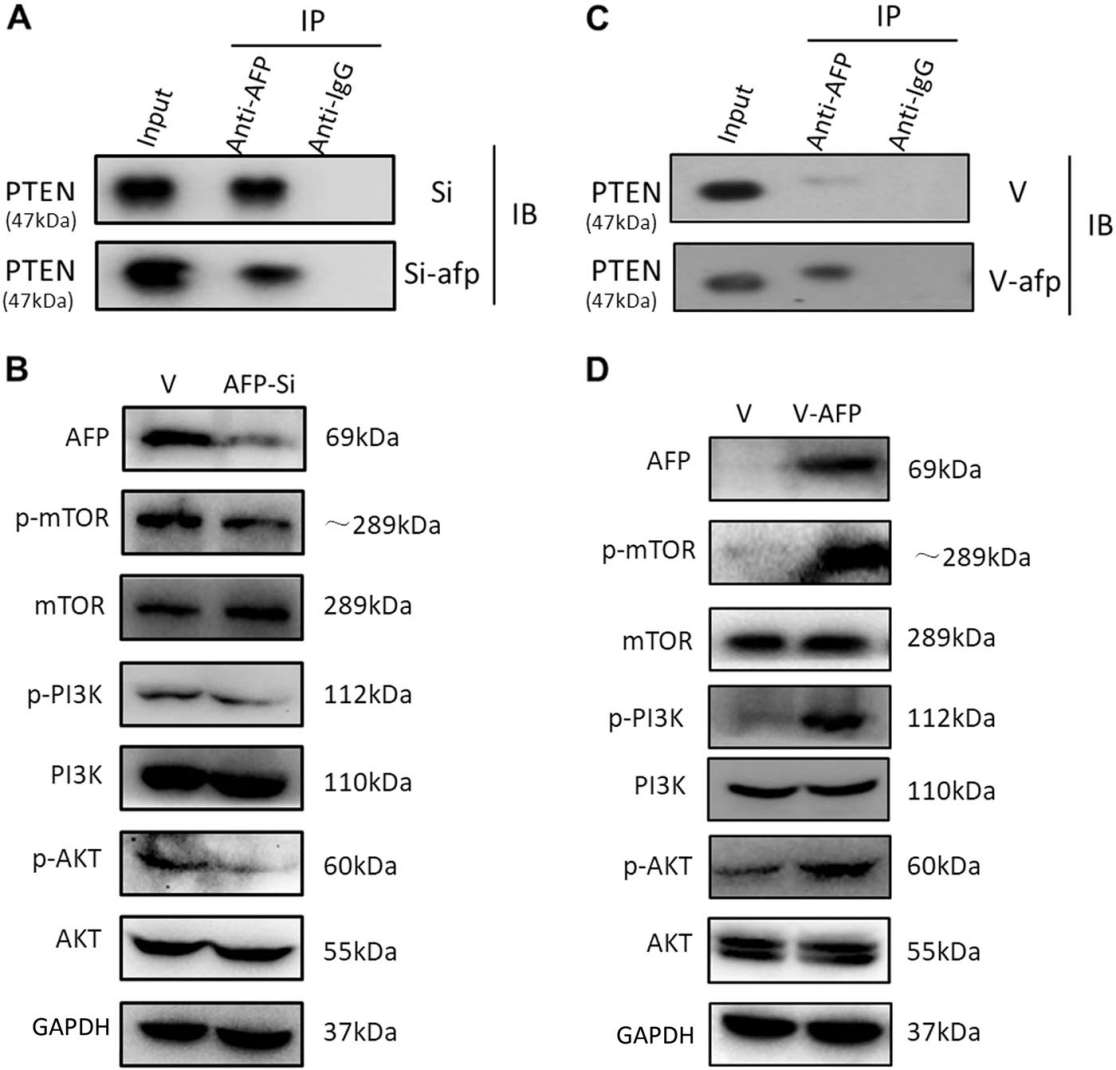
Expression of PI3K/AKT/mTOR signalling-related proteins after AFP knockdown or *afp* gene transfection in PLC/PRF/5 and HLE cells. **a** Co-immunoprecipitation analysis of the interaction between AFP and PTEN in AFP-siRNA923 (AFP-si)-transfected PLC/PRF/5 cells. **b** Western blotting for the effects of AFP knockdown on the expression of PI3K/AKT/mTOR signalling-related proteins in AFP-si-transfected cells. **c** Co-immunoprecipitation analysis of the interaction between AFP and PTEN in pcDNA3.1-afp (V-afp)-transfected HLE cells. **d**. Western blotting for the effects of AFP overexpression on the expression of PI3K/AKT/mTOR signalling-related proteins in HLE cells. Images are representative of at least three independent experiments

PTEN is a critical molecule that inhibits PI3K/Akt signalling^18^. We subsequently assessed the impact of AFP on this signalling pathway in HCC cells. CoIP and Western blotting showed that following AFP knockdown with si-afp in PLC/PRF/5 cells, the interaction between AFP and PTEN was reduced (Fig. 2a). The core components of the active PI3K-Akt-mTOR signalling pathway, namely, p-Akt, p-PI3K and p-mTOR, were markedly decreased by AFP knockdown (Fig. 2b). When AFP was overexpressed in HLE cells by transfection with v-afp, AFP interacted with PTEN, as detected by CoIP (Fig. 2c). Western blotting also demonstrated that AFP overexpression significantly increased p-Akt, p-PI3K and p-mTOR levels in HLE cells (Fig. 2d).

### AFP inhibited HCC cell autophagy by upregulating p-mTOR

**Fig. 3:**
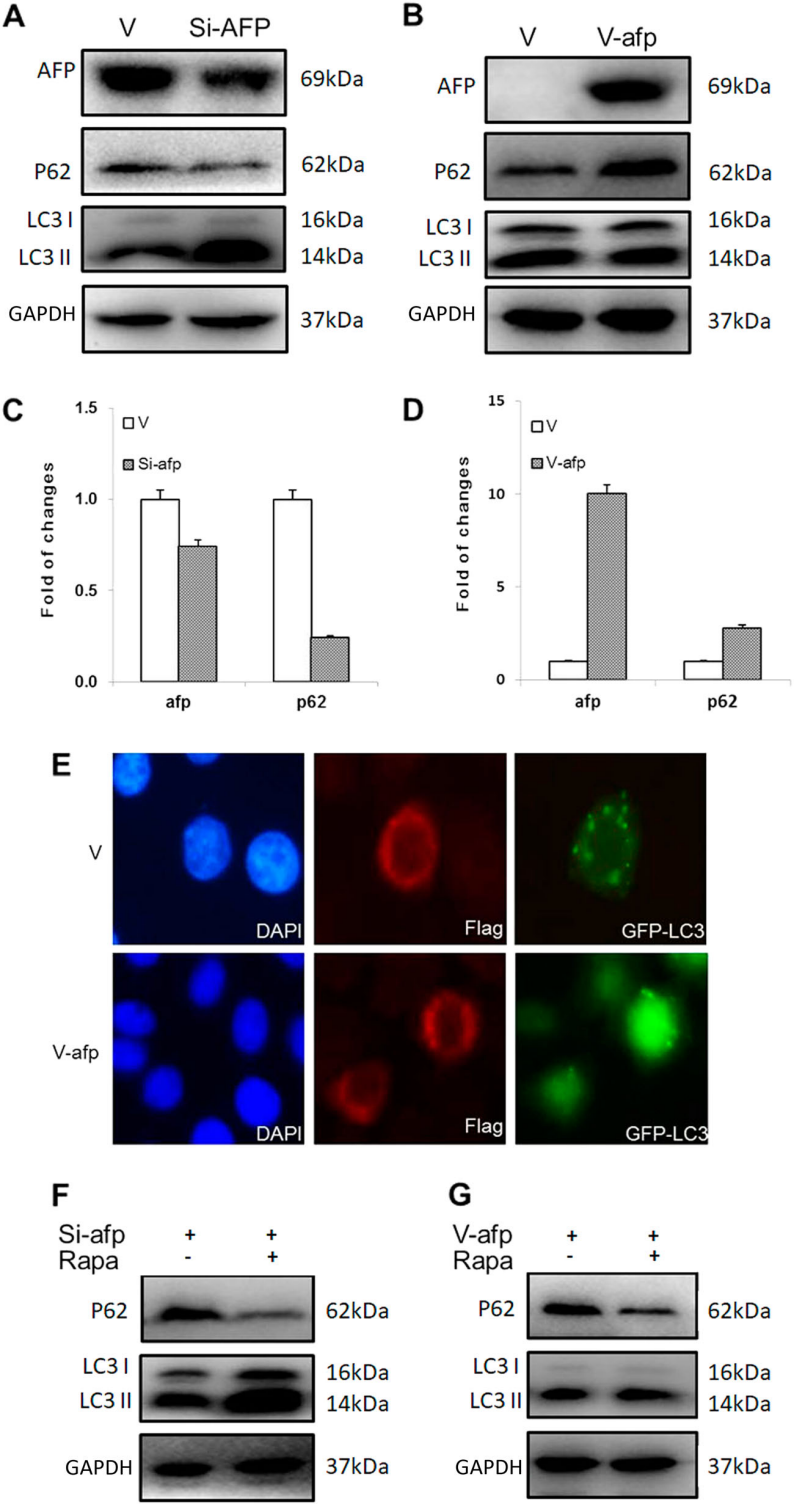
The effect of AFP on autophagy in PLC/PRF/5 and HLE cells. Effects of AFP on P62 and LC3 gene expression in AFP-si-transfected PLC/PRF/5 cells analysed by Western blotting (**a**) and RT-qPCR (**c**). Western blotting (**b**) and RT-qPCR (**d**) were performed to analyse the effect in V-afp-transfected HLE cells. **e** HLE cells were transfected with V-afp and GFP-LC3 plasmids for 24 h, and AFP (red) and LC3 puncta (green) were observed via fluorescence microscopy. Nuclei (blue) were counterstained with DAPI. Western blotting was performed to detect P62 and LC3 expression in AFP-si-transfected PLC/PRF/5 (**f**) and V-afp-transfected HLE (**g**) cells treated with rapamycin (Rapa). Images are representative of three independent experiments

Since PI3K-Akt-mTOR signalling is closely associated with autophagy^19,20^, we examined the relationship between AFP levels and the expression of the autophagy-related proteins LC3 and P62. Western blotting and RT-qPCR analyses showed that in AFP-knockdown PLC/PRF/5 cells, P62 expression was decreased, whereas LC3II expression was significantly enhanced (Figs. 3a, c). In contrast, AFP-overexpressing HLE cells displayed enhanced P62 expression and decreased LC3II expression (Figs. 3b, d). We further observed the formation of autophagosomes in HLE cells using fluorescence microscopy. As shown in Fig. 3e, cells overexpressing both AFP and GFP-LC3 exhibited fewer fluorescent puncta than cells lacking AFP expression, indicating decreased autophagy. The ability of AFP to decrease cell autophagy was impacted by rapamycin, a specific inhibitor of p-mTOR. Western blotting revealed that rapamycin further enhanced the LC3II upregulation and P62 downregulation associated with AFP silencing in PLC/PRF/5 cells (Fig. 3f), restored the inhibition of LC3II expression by AFP and stimulated the expression of p62 in HLE cells (Fig. 3g).

### AFP stimulated HCC cell growth

**Fig. 4:**
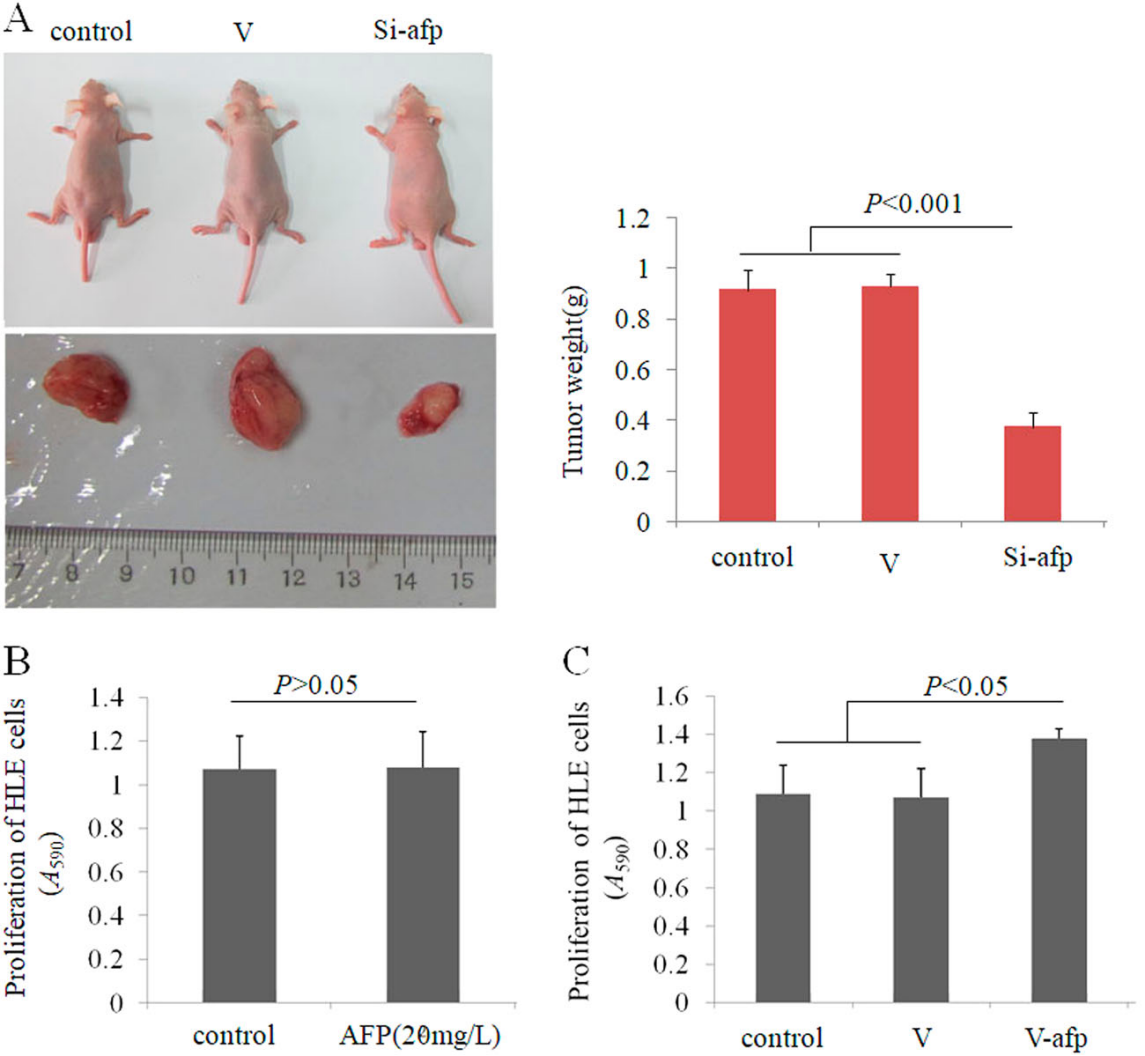
The effect of AFP on HCC cell growth in vivo and in vitro. **a** PLC/PRF/5 cells were transfected with control-siRNA vector (V) or AFP-siRNA923, and PLC/PRF/5 cells (1 × 10^6^) in 0.1 mL of Hank balanced salt solution were then subcutaneously injected into the right scapular region of male nude mice (*n* = 10). Tumour-bearing mice were killed when they became moribund on day 30 after inoculation, and tumours were removed and weighted. The right column graph indicates the tumour weight. **b** HLE cells were treated with AFP (20 mg/L) for 48 h. **c** HLE cells were transfected with pcDNA3.1-afp vector and cultured for 48 h, and MTT assays were performed to detect cell proliferation (*n* = 6)

To observe the effect of AFP on the growth of HCC cells, we performed experiments with animal models. PLC/PRF/5 cells (AFP-producer) were transfected with AFP-siRNA vector and subcutaneously injected into nude mice. Tumour-bearing mice were killed, and their tumours were removed and weighed. According to the results, inhibiting AFP expression significantly decreased tumour weight (Fig. 4a). We also applied MTT assays to analyse the effect of AFP on HLE cell proliferation in vitro. HLE cells were cultured in media supplemented with AFP or transfected with the AFP-overexpression vector pcDNA-*afp*. Although AFP supplementation in the media did not influence HLE cell growth (Fig. 4b), HLE cell proliferation was significantly stimulated by transfection with the pcDNA-*afp* vector. These results demonstrated that AFP can promote HCC cell growth both in vivo and in vitro.

### AFP inhibited apoptosis and promoted migration in HCC cells

**Fig. 5:**
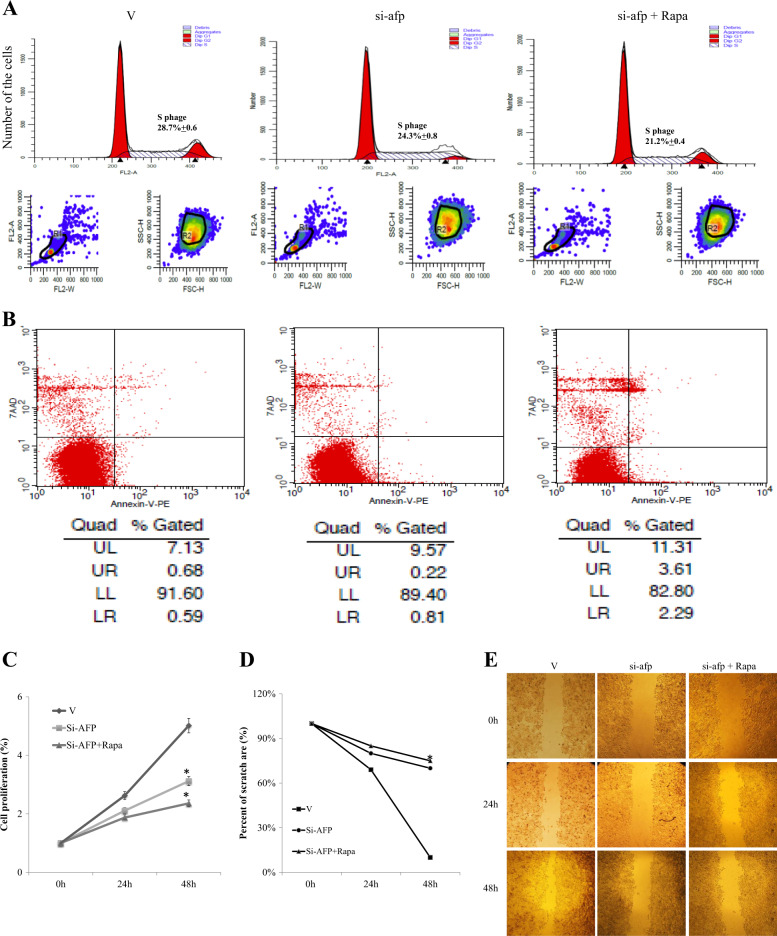
Effects of AFP and rapamycin on PLC/PRF/5 cell proliferation and migration. PLC/PRF/5 cells were transfected with control-siRNA vector (V) or AFP-siRNA923, followed by treatment with 10 nM rapamycin. The resulting cell cycle distribution (**a**) and proportion of apoptotic cells (**b**) were determined by flow cytometry. **c** CCK-8 assays were used to monitor cell proliferation under different treatments at 0, 24 and 48 h. Scratch tests were performed to detect cell migration under different treatments, and images were acquired on a microscope at 0, 24 and 48 h (**e**). **d** Cell migration ratios are shown in the histogram. Images are representative of at least three independent experiments

**Fig. 6:**
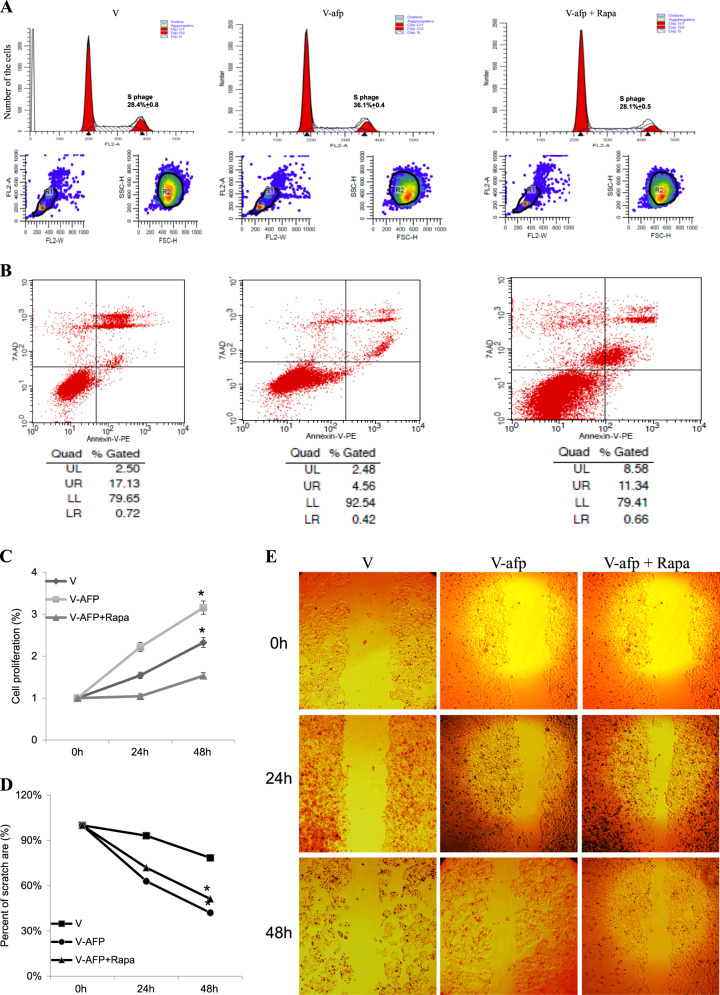
Effects of AFP and rapamycin on HLE cell proliferation and migration. HLE cells were transfected with control vector (V) or pcDNA3.1-afp (V-afp), followed by treatment with rapamycin (Rapa). The resulting cell cycle distribution (**a**) and population of apoptotic cells (**b**) were determined by flow cytometry. **c** CCK-8 assays were used to monitor cell proliferation under different treatments at 0, 24 and 48 h. Scratch tests were performed to detect cell migration (**e**). **d** Cell migration ratios are shown in the histogram. Images are representative of at least three independent experiments

We also investigated the impacts of AFP expression on the cell cycle distribution, apoptosis, proliferation and migration of HCC cells. FCM analysis showed that in PLC/PRF/5 cells, AFP gene knockdown reduced the percentage of cells in S phase, and this effect was further amplified by treatment with rapamycin (Fig. 5a). In HLE cells, AFP overexpression increased the fraction of cells in S phase, and this effect was inhibited by rapamycin treatment (Fig. 6a). The percentage of apoptotic cells was significantly increased following AFP gene knockdown in PLC/PRF/5 cells (Fig. 5b), and rapamycin administration further enhanced AFP knockdown-mediated apoptosis. In contrast, AFP overexpression reduced apoptosis of HLE cells, and this effect was abolished by rapamycin treatment (Fig. 6b). Furthermore, CCK-8 assays showed that AFP knockdown in PLC/PRF/5 cells inhibited cell proliferation, while AFP overexpression in HLE cells promoted cell growth. However, AFP-associated cell proliferation was inhibited by the administration of rapamycin (Figs. 5c and 6c). Next, the effects of AFP and rapamycin on cell migration were evaluated with scratch tests. As shown in Figs. 5e and 6e, the cell migration ability was decreased by siRNA-mediated AFP gene knockdown in PLC/PRF/5 cells (Fig. 5d-e) but was significantly increased when AFP was overexpressed in HLE cells (Fig. 6d-e). AFP-related migration was also inhibited by rapamycin in both cell lines (Figs. 5e and 6e).

### AFP promoted the invasion of HCC cells in vitro

**Fig. 7:**
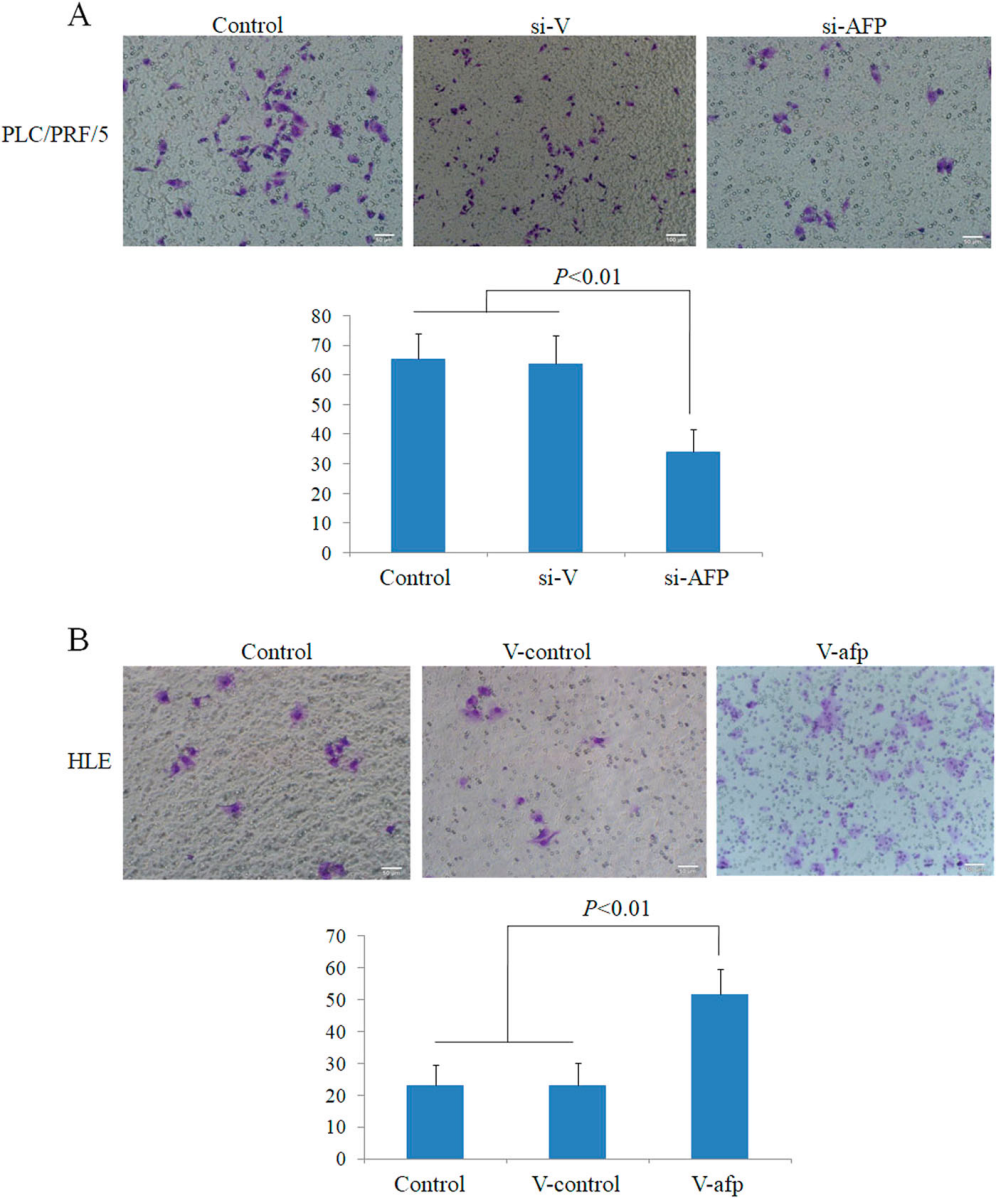
Effects of AFP on the invasion of PLC/PRF/5 and HLE human liver cancer cells. **a** PLC/PRF/5 cells were transfected with AFP-siRNA vectors for 48 h, and invasive cells were stained with 0.1% crystal violet and observed by microscopy; the lower column graph indicates the quantity of invasive cells. *P* < 0.01 versus control and scrambled-siRNA groups. **b** HLE cells were transfected with pcDNA3.1-afp for 48 h, and invasive cells were stained with 0.1% crystal violet and observed by microscopy; the lower column graph indicates the quantity of invasive cells. *P* < 0.01 versus control and pcDNA3.1-vector groups. Three independent experiments were performed to generate these data

We then performed a transwell chamber assays to examine whether AFP influences invasion by HCC cells. The invasion assays indicated that the capacity of PLC/PRF/5 cells to migrate through the pores was significantly decreased upon transfection with si-afp for 48 h compared with control cells and cells transfected with scrambled siRNA (*P* < 0.05) (Fig. 7a). However, the migratory capacity of HLE cells was significantly enhanced upon transfection with v-afp for 48 h, in contrast to control cells and cells transfected with empty vector (*P* < 0.05) (Fig. 7b). These results indicated that AFP has the ability to promote HCC cell invasion in vitro.

## Discussion

The propensity of HCC to undergo highly malignant growth, invasion, metastasis and recurrence has made it the second most-frequent cause of cancer-related death in China^21^. AFP has served as a classical biomarker of liver cancer since the 1970s but is now recognized as a key signalling molecule involved in several aspects of tumour biology^13–15,22,23^. The data gathered in the present study suggest that AFP can regulate HCC cell autophagy by promoting PI3K/Akt/mTOR signalling by binding to PTEN.

Although our previous work demonstrated that cytoplasmic AFP was able to co-localize and interact with PTEN in the HepG2 and Bel7402 HCC cell lines^13–15^, the clinical significance of this finding, particularly the influence on cell autophagy, remained unknown. In the present study, the PLC/PRF/5 and HLE cell lines were used to study the relationship between AFP and autophagy. Our data showed that AFP was able to interact with PTEN in the cytoplasm of PLC/PRF/5 cells and of pcDNA3.1-afp-transfected HLE cells. These results imply a potential functional role for these proteins in a complex signalling network.

PTEN is known to be a tumour suppressor involved in the regulation of cell proliferation, survival, migration, invasion and angiogenesis^24^. PTEN has been shown to block PI3K/AKT signalling by inhibiting the activation of AKT^25^, and the loss or mutation of PTEN may lead to AKT activation and tumour development^26,27^. Our study showed that AFP overexpression significantly increased PI3K-AKT-mTOR signalling pathway activity in pcDNA3.1-afp-transfected HLE cells^13–15^, whereas AFP knockdown markedly decreased the expression of the core components of the PI3K-AKT-mTOR signalling pathway in AFP-siRNA923-transfected PLC/PRF/5 cells. Abnormal AKT activation plays a critical role in the development of cancer, but growing evidence suggests that PI3K-AKT-mTOR signalling is important in cell autophagy^28,29^. Therefore, AFP appears to be a component of the PI3K/Akt/mTOR signalling pathway that is involved in regulating hepatocellular autophagy.

In most situations, autophagy has been recognized as a survival-promoting pathway that facilitates tumourigenesis by increasing cancer cell growth and aggressiveness in anoxic and glucose-deprived microenvironments^28^. In the present study, the effect of AFP on autophagy was apparent in PLC/PRF/5 and HLE cells. This effect was achieved through the binding of AFP to PTEN, thereby inducing PI3K/Akt/mTOR signalling activation. To further explore the biological functions of this effect, we analysed cell cycle, apoptosis, proliferation and migration under different treatment conditions. In PLC/PRF/5 cells, AFP abolished the induction of apoptosis, inhibited the cell cycle and proliferation, and blocked cell migration, which was further amplified by treatment with rapamycin. Rapamycin is recognized as an autophagy agonist that specifically inhibits mTOR. This effect of AFP was confirmed in HLE cells transfected with the AFP gene. Together, these results clearly showed that AFP affects HCC cell autophagy through mTOR.

Collectively, our results suggest a functional link between AFP and mTOR-induced cell autophagy, providing evidence to further understand the novel role of cytoplasmic AFP in cell growth. Over the last decade, multiple studies have shown that specific inhibitors of mTOR can inhibit liver cancer growth^26^ and that targeting AKT and mTOR is able to inhibit the proliferation of HCC cells^30^. AFP plays a critical role in promoting HCC cell metastasis through activation of the PI3K/AKT signalling pathway^31^. The absence of AFP from the tumour environment has been shown to greatly reduce tumour lethality and HCC growth, whereas the presence of AFP increases tumour lethality^32^. In the present study, we found that AFP was able to stimulate HCC cell growth in vivo and vitro. Previously, we found that HLEs lack expression of the AFP receptor^33^ and that adding AFP to the culture media did not play a role in enhancing the growth of HLE cells; however, AFP was able to stimulate proliferation of HLE cells transfected with an AFP-overexpression vector, demonstrating that cytoplasmic AFP may activate growth signals to stimulate HCC cell growth. AFP also antagonizes anticancer agents that inhibit the malignant behaviours of HCC cells^34^. These results imply that AFP is a critical factor for promoting the malignant behaviours of HCC cells. Considering the effects of AFP on mTOR expression, a potential treatment strategy may involve a combination of AFP gene silencing or depletion of circulating AFP and treatment with specific mTOR inhibitors to enhance the therapeutic potential of targeted mTOR treatment. In conclusion, AFP promotes malignant behaviour by inhibiting HCC cell autophagy, and the likely mechanism may involve the activation of the PI3K/AKT/mTOR signalling pathway. Silencing AFP expression and suppressing mTOR activity are novel strategies for the treatment of HCC patients.

## Materials and methods

### Cells

The HCC cell lines PLC/PRF/5 and HLE were kindly provided by Dr Gang Li (Department of Biochemistry and Molecular Biology, Peking University). Both cell lines were maintained in a 5% CO_2_ incubator in DMEM supplemented with 10% FCS.

### Western blotting

Western blotting was performed as previously described^16^. Briefly, total cellular proteins were extracted using radioimmunoprecipitation (RIPA) assay buffer containing protease inhibitors. Thirty micrograms of protein was used for Western blotting. The signal was visualized by incubation with the Enhanced Chemiluminescence Kit (Millipore Co. USA) and recorded on a FUJI LAS 3000 imaging system (Japan). All primary and secondary antibodies for Western blotting are listed in Table 1.

**Table 1:** Antibodies and sequences used for Western blotting and RT-qPCR. And sequences of siRNA923 and scrambled siRNA

Protein	Mfrs. /Made in	Cat No.
*Primary antibodies*		
AFP	Santa Cruz BiotechInc./USA	sc-8399
PTEN	Cell Signaling Technology/USA	9188S
LC3	Sigma/USA	L7543-100UL
PI3K	Cell Signaling Technology/USA	4249P
AKT	Cell Signaling Technology/USA	2920P
mTOR	Cell Signaling Technology/USA	2983P
p-PI3K	Cell Signaling Technology/USA	4228P
p-AKT	Cell Signaling Technology/USA	4060P
p-mTOR	Cell Signaling Technology/USA	5536P
P62	Cell Signaling Technology/USA	5114S
Flag	Sigma/USA	F7425
GAPDH	Sigma/USA	G9545
*Secondary antibodies*		
HRP-conjugated anti-mouse IgG	Zhongshan Boil Tech Co/China	IH-0031
HRP-conjugated anti- rabbitIgG	Zhongshan Boil Tech Co/China	IH-0011
Alexa Fluor 568	Thermo Fisher Scientific/USA	A-11004
Alexa Fluor 488	Thermo Fisher Scientific/USA	A-11034
Gene	Primer sequences	Product size (bp)
	RT-qPCR	
P62	Sense	5′-GGTGGCACTTGTTATGCTATCCT-3'75
	Antisense 5′-GCCCATGGGAACTCACAATC-3′	
AFP:	Sense	5′-CCAACAGGAGGCCATGCTT-3'61
	Antisense	5′-GAATGCAGGAGGGACATATGTTT-3′
GAPDH	Sense	5′-TGAAGGTCGGAGTCAACGGA-3'232
	Antisense	5′-CCTGGAAGATGGTGATGGGAT-3′
AFP	SiRNA	
SiRNA923	Sense	5′-CACCGAACGTGGTCAATGTATAATTCAAGAGATTATACATTGACCACGTTCTTTTTTG-3′
	Antisense	5′-GATCCAAAAAAGAACGTGGTCAATGRATA
		ATCTCTTGAATTATACATTGACCACGTTC-3′
Scrambled siRNA	Sense	5′-CACCGTTCTCCGAACGTGTCACGTCAAGA
		GATTACGTGACACGTTCGGAGAATTTTTG-3′
	Antisense	5′-GATCCAAAAAATTCTCCGAACGTGTCA
		GTCTCTTGACGTGACACGTTCGGAGAAC-3′

### Immunofluorescence

Cells were seeded on coated glass coverslips and then fixed in 4% paraformaldehyde solution. Rabbit anti-PTEN and mouse anti-AFP antibodies were added, and the slides were incubated overnight at 4 °C. Cells were incubated with Alexa Fluor 594 and 488 secondary antibodies (Thermo Fisher Scientific) for 1 h at room temperature, followed by DAPI counterstaining. Cell images were captured on a laser scanning confocal microscope (Leica TCSSTED-3×). FRET experiments were performed on a laser scanning confocal microscope (Leica TCSSTED-3×). The pre- and post-bleaching fluorescence intensity changes are shown as percentages (efficiency, E). Three independent experiments were performed. At least 10 cells were analysed in each experiment.

### Transient transfection

The construction of plasmids expressing AFP (pcDNA3.1-afp, V-afp) and AFP-siRNA923 (si-afp) was described previously^13^. Plated cells were transfected at 80% confluence. The plasmids were diluted and mixed with the transfection reagent according to the manufacturer’s instructions. The cells were incubated with the plasmid-lipid complex for 24 h.

### Co-immunoprecipitation (CoIP)

The interaction of AFP with PTEN in HCC cells was evaluated by CoIP. Cells were lysed in RIPA buffer containing 1% protease inhibitors. The lysates (2 mg/mL) were incubated overnight with 1 µg of anti-AFP, anti-IgG or anti-PTEN antibody at 4 °C, followed by incubation with 100 µl Protein A Sepharose (CL-4B) (17-0780-01, GE) for an additional 8 h. The beads were collected and washed 3 times with 1 mL PBS before being boiled in loading buffer. Western blotting was used to analyse the CoIP results.

### Quantitative real-time reverse transcription PCR (RT-qPCR)

Quantitative RT-qPCR assays were used to evaluate P62 and GAPDH expression. Primer sequences are listed in Table 1. Total cellular RNA was isolated using the TRIzol reagent (15596026, Thermo Fisher Scientific) and subsequently subjected to reverse transcription (AT301-03, TransGen Biotech). RT-qPCR was performed using the SYBR (QPK-201, TOYOBO) method on an ABIV7 machine.

### Animal experiments

Male pathogen-free athymic nude mice were purchased from the Guangzhou Animal Research Center (Guangzhou, China). The animals were maintained in facilities approved by the Ethical Committee of Hainan Medical College. Investigation procedures were approved by Hainan Medical College Institutional Committee. To analyse tumourigenicity, PLC/PRF/5 HCC cancer cells (1 × 10^6^) in 0.1 mL of Hank’s balanced salt solution were subcutaneously injected into the right scapular region of nude mice (10 per group). Tumour-bearing mice were sacrificed when they became moribund on day 30 after inoculation, and their tumours were removed and weighted.

### MTT analysis of HCC cell proliferation

To determine the effect of AFP on the proliferation of HLE cells, the cells were transfected with pcDAN3.1-afp for 24 h and then plated at a density of 1.5 × 10^4^ cells per well in 96-well plates and cultured in DMEM medium supplemented with 10% FCS at 37 °C in a humidified atmosphere with 5% CO_2_ for 48 h. Then, the supernatants of the cultured cells were replaced with medium lacking FCS for another 24 h. The non-transfected cells were treated with AFP (Sigma, USA) concentrations (20 mg/L) for 48 h. The effects of AFP on cell growth were measured using the methylthiazolyldiphenyltetrazolium bromide (MTT) assay as previously described^34^ and following a standard procedure.

### Flow cytometry (FCM)

An Annexin V FITC Apoptosis Detection Kit (AD10, Dojindo) was used to evaluate cell apoptosis. Briefly, cells exposed to different treatments were resuspended in diluted Annexin V binding solution to a final cell concentration of 1 × 10^5^ cells/100 µL. Then, the cells were incubated with 5 µL of FITC-conjugated Annexin V and 5 µL of PI for 15 min at room temperature in the dark. Before flow cytometry, the above solution was diluted to 500 µL with Annexin V binding solution. The percentage of apoptotic cells was calculated as the number of Annexin V(+)/PI(−) cells divided by the number of Annexin V(+)/PI(+) cells.

Cell cycle distribution was evaluated by FCM following PI staining, as previously described^16^. PLC/PRF/5 and HLE cells were transfected with si-afp and V-afp and then treated with 10 nM rapamycin for 24 h. A FACScan-420 flow cytometer (Becton-Dickinson, USA) was used to measure cellular DNA content.

### Determination of viability

The Cell Counting Kit (CCK)-8 (CK04, Dojindo) was used to assess the effect of AFP and rapamycin on cell proliferation. PLC/PRF/5 and HLE cells were seeded into 96-well microplates with different treatments (vector, si-afp/V-afp and 10 nM rapamycin). The absorbance at 570 nm was measured on a Universal Microplate Reader (EL x800). The percentage of cell viability was calculated as [(*A*_570sample-background_)/(*A*_570control-backgroud_)] × 100%.

### Scratch test

PLC/PRF/5 and HLE cell migration was evaluated using scratch tests. Cells were grown to 80% confluence in 12-well microplates before being scratched. Cell images were captured on a light microscope at 0, 24 and 48 h following treatment. The scratch area was calculated using ImageJ software.

### Cell invasion assay

Cell invasion assays were carried out according to the manufacturer’s protocols. To measure cell invasion, transwell chambers were used to hold inserts containing cultured cells (Transwell chamber; 8-mm pore size; Costar, High Wycombe, UK). PLC/PRF/5 and HLE cells were placed into the wells of 12-well culture plates, and the upper and lower chambers were separated. The cells (5 × 10^4^) were added to the upper chamber and cultured with serum-free DMEM, whereas the lower chamber was filled with complete medium containing 20% FCS. After 48 h of incubation, the cells in the upper chamber were carefully removed with a cotton swab, and those that had migrated through the membrane to the lower surface were fixed in 90% methanol and stained with 0.1% crystal violet. The number of cells that had migrated through the pores was quantified by counting five independent visual fields on a microscope (Olympus) using a 20× objective. Three independent assays were performed.
