# Author Correction: Alpha-fetoprotein inhibits autophagy to promote malignant behaviour in hepatocellular carcinoma cells by activating PI3K/AKT/mTOR signalling

**DOI:** 10.1038/s41419-019-1302-1

**Published:** 2019-01-28

**Authors:** Shanshan Wang, Mingyue Zhu, Qiaoyun Wang, Yuli Hou, Lei Li, Honglei Weng, Yan Zhao, Dexi Chen, Huiguo Ding, Junli Guo, Mengsen Li

**Affiliations:** 1 Hainan Provincial Key Laboratory of Carcinogenesis and Intervention, Hainan Medical College, Haikou, China; 2 Beijing Institute of Hepatology, Beijing Youan Hospital, Capital Medical University, Beijing, China; 3 Beijing Precision Medicine and Transformation Engineering Technology Research Center of Hepatitis and Liver Cancer, Beijing, China; 4 Clinical Laboratory Center, Beijing Youan Hospital, Capital Medical University, Beijing, China; 5 Department of Gastrointestinal and Hepatology, Beijing Youan Hospital, Capital Medical University, Beijing, China; 6 Molecular Hepatology, University of Heidelberg, University Medical Center Mannheim, 68167 Mannheim, Germany

**Correction to:** **Cell Death and Disease** (2018)9:1027

10.1038/s41419-018-1036-5

published online 09 October 2018

The correct ordering and designations for corresponding/first authors are as follows:

Shanshan Wang^1,2*^, Mingyue Zhu^3^, Qiaoyun Wang^3^, Yuli Hou^4^, Lei Li^5^, Honglei Weng^6^, Yan Zhao^4^, Dexi Chen^1,2^, Junli Guo^3^, Huiguo Ding^#5^, Mengsen Li^#3^

^1^Beijing Institute of Hepatology, Beijing Youan Hospital, Capital Medical University, Beijing, China

^2^Beijing Precision Medicine and Transformation Engineering Technology Research Center of Hepatitis and Liver Cancer, Beijing, China

^3^Hainan Provincial Key Laboratory of Carcinogenesis and Intervention, Hainan Medical College, Haikou, China

^4^Clinical Laboratory Center, Beijing Youan Hospital, Capital Medical University, Beijing, China

^5^Department of Gastrointestinal and Hepatology, Beijing Youan Hospital, Capital Medical University, Beijing, China

^6^Molecular Hepatology, University of Heidelberg, University Medical Center Mannheim, 68167 Mannheim, Germany

*First author

# Corresponding author.

