# Publisher Correction: Alpha-fetoprotein inhibits autophagy to promote malignant behaviour in hepatocellular carcinoma cells by activating PI3K/AKT/mTOR signalling

**DOI:** 10.1038/s41419-018-1292-4

**Published:** 2019-03-01

**Authors:** Shanshan Wang, Mingyue Zhu, Qiaoyun Wang, Yuli Hou, Lei Li, Honglei Weng, Yan Zhao, Dexi Chen, Junli Guo, Huiguo Ding, Mengsen Li

**Affiliations:** 1 Beijing Institute of Hepatology, Beijing Youan Hospital, Capital Medical University, Beijing, China; 2 Beijing Precision Medicine and Transformation Engineering Technology Research Center of Hepatitis and Liver Cancer, Beijing, China; 3 Hainan Provincial Key Laboratory of Carcinogenesis and Intervention, Hainan Medical College, Haikou, China; 4 Clinical Laboratory Center, Beijing Youan Hospital, Capital Medical University, Beijing, China; 5 Department of Gastrointestinal and Hepatology, Beijing Youan Hospital, Capital Medical University, Beijing, China; 6 Molecular Hepatology, University of Heidelberg, University Medical Center Mannheim, 68167 Mannheim, Germany

**Correction to:** **Cell Death and Disease;**

10.1038/s41419-018-1036-5;

published online 9 October 2018

The article published with errors in the authors’ information. The correct ordering and designations for corresponding /first authors are shown here.

